# Neutrophil Extracellular Trap Induced Dendritic Cell Activation Leads to Th1 Polarization in Type 1 Diabetes

**DOI:** 10.3389/fimmu.2020.00661

**Published:** 2020-04-14

**Authors:** Zuzana Parackova, Irena Zentsova, Petra Vrabcova, Adam Klocperk, Zdenek Sumnik, Stepanka Pruhova, Lenka Petruzelkova, Robert Hasler, Anna Sediva

**Affiliations:** ^1^Department of Immunology, 2nd Faculty of Medicine Charles University, University Hospital in Motol, Prague, Czechia; ^2^Department of Pediatrics, 2nd Faculty of Medicine Charles University, University Hospital in Motol, Prague, Czechia; ^3^Institute of Clinical Molecular Biology, University Hospital in Schleswig-Holstein, Kiel, Germany; ^4^Christian-Albrecht University of Kiel, Kiel, Germany

**Keywords:** NET, netosis, type 1 diabetes, monocyte-derived dendritic cells, innate immunity, autoimmunity, neutrophils

## Abstract

Neutrophils releasing neutrophil extracellular traps (NETs) infiltrate the pancreas prior to type 1 diabetes (T1D) onset; however, the precise nature of their contribution to disease remains poorly defined. To examine how NETs affect immune functions in T1D, we investigated NET composition and their effect on dendritic cells (DCs) and T lymphocytes in T1D children. We showed that T1D patient NET composition differs substantially from that of healthy donors and that the presence of T1D-NETs in a mixed peripheral blood mononuclear cell culture caused a strong shift toward IFNγ-producing T lymphocytes, mediated through activation of innate immunity cells in T1D samples. Importantly, in a monocyte-derived DC (moDC) culture, NETs induced cytokine production, phenotypic change and IFNγ-producing T cells only in samples from T1D patients but not in those from healthy donors. RNA-seq analysis revealed that T1D-NETs presence causes TGFβ downregulation and IFNα upregulation and creates pro-T1D signature in healthy moDCs.

## Introduction

Type 1 diabetes (T1D) is generally viewed as a Th1-mediated autoimmune disease in which cytotoxic T lymphocytes attack insulin-producing beta cells in pancreatic islets. However, infiltrating autoreactive T lymphocytes represent only one part of a more complex network of immune cells located in the pancreatic islets during the development of T1D. Macrophages, dendritic cells (DC) and natural killer cells have all been well documented to play crucial roles in T1D pathogenesis (1). Neutrophils infiltrate the islets prior to the onset of T1D symptoms as well as during disease progression (2), but the precise role of neutrophils, which are the most plentiful leukocytes and first responders during inflammation, remains elusive (3).

Apart from the classic defense mechanisms that neutrophils possess, such as phagocytosis, reactive oxygen species (ROS) production and granule degranulation, neutrophils might also form extracellular networks known as NETs (neutrophil extracellular traps). They are released to trap, neutralize, and kill bacteria (4), viruses (5) and fungi (6). NETs are large, extracellular, web-like structures composed of cytosolic and granule proteins that are assembled on decondensed chromatin (4). Notably, NETs represent endogenous danger signals that are sufficient to initiate inflammation in the absence of microbial priming and can potentiate the resolution of inflammation. NETs escalate inflammation through the regulation of inflammatory cytokines directly or indirectly by modulating and activating other immune cells (7, 8).

However, NETs are also a source of autoantigens and can contribute to the pathogenesis of some autoimmune diseases, particularly those associated with autoantibodies against neutrophil-derived proteins such as antineutrophil cytoplasmic antibody (ANCA) – associated vasculitis (9). In systemic lupus erythematosus (SLE), NETs can activate plasmacytoid dendritic cells (pDCs) and trigger type I interferon (IFN) production and drive autoimmune pathology (10, 11).

Little is known about the involvement of NETs in T1D. Although controversial opinions emerged about the presence of NET-related biomarkers in the peripheral blood at T1D (12, 13), neutrophils releasing NETs have been clearly shown to be present in the pancreas of pre-symptomatic and symptomatic T1D patients, and it is thus safe to assume they may play a role in the initiation of beta cell destruction (2, 14). In addition, high glucose levels were reported to impair and delay NET release in diabetic patients, providing a contributing mechanism for the increased susceptibility to infections and wound healing impairment of diabetic patients (15, 16). In contrast, other reports have shown that neutrophils from patients with diabetes release NETs more readily, which may be linked to an elevated glucose level (17–19).

Therefore, we initiated a study of NETs in pediatric T1D patients, aiming to evaluate the differences in NET composition, their effect on antigen presenting cell phenotype, cytokine production and subsequent induction of T cell differentiation.

## Patients and Methods

### Patients

Written informed consent was obtained from all the patients or the patients’ parents/guardians in accordance with the Declaration of Helsinki, and the study was approved by the Ethics Committee of University Hospital Motol.

A cohort of 45 pediatric patients with T1D (59% female) and 30 healthy donors (48% female) was included in this study. The median age of patients with T1D was 15.5 ± 2 years (range: 11.2–18.6 years) and of the healthy donors was 18.5 ± 2.9 years (range: 12.2–21.6 years). All patients with T1D were treated with insulin since disease onset. The median T1D duration was 6.7 ± 3.7 years (range: 1.1–15.3 years). The median of their last glycated hemoglobin (HbA1c) was 61 ± 12 mmol/mol (range: 39–87 mmol/mol). At the time of blood sampling, patients were metabolically stable; none of them had signs of active infection, neoplasia or other comorbidities except well-controlled celiac disease or autoimmune thyroiditis (11% of recruited patients with T1D). The healthy donors had a negative personal history of autoimmune diseases.

### Cell Isolation and Culture

Peripheral blood was collected from patients and healthy volunteers into EDTA-coated tubes. First, peripheral blood mononuclear cells (PBMCs) were isolated using Ficoll-Paque (GE Healthcare Bio-Sciences, Uppsala, Sweden). Neutrophils were further isolated using the Dextran sedimentation method, remaining red blood cells were hypotonically lysed and granulocytes were washed twice in PBS without EDTA. Neutrophil purity was < 95%, the major contaminants were eosinophils. The obtained cells were resuspended in RPMI 1640 medium with sodium bicarbonate buffer system, supplemented with 2% autologous serum, 1% penicillin and streptomycin and 1% Glutamax (Thermo Fisher Scientific, Waltham, MA, United States).

### NET Induction

NET formation was induced according a protocol adapted from (7). Briefly, the isolated neutrophils were plated in 6-well plates at a density of 10^6^ cells/ml and stimulated with 50 nM phorbol myristate acetate (PMA) (20, 21) (Cayman Chemicals, Ann Arbor, United States) for 3 h in 37°C in 5% CO_2_ to maintain physiological pH. The culture medium was then carefully removed; the NETs were carefully washed with PBS to remove possible products of neutrophil activation or degranulation and then partially digested by a restriction enzyme mix containing enzymes AluI, NdeI and PacI (all from Thermo Fisher Scientific) at a concentration of 10 U/ml in complete media supplemented with 2% autologous serum. The digest was performed at 37°C for 20 min, followed by centrifugation at 12,000 × *g* for 10 min to remove cells and debris. The NET-DNA concentration was determined on a Nanodrop (Thermo Fisher Scientific). NET-containing supernatants were then characterized for their DNA and protein content using several approaches. The size of the DNA fragments was analyzed by electrophoresis. For western blot analysis, membranes with loaded samples were incubated with the primary antibodies anti-PR3 (Abcam, Cambridge, United Kingdom) overnight, followed by incubation with peroxidase-conjugated anti-rabbit or anti-mouse secondary antibodies for 2 h. The membranes were developed using SuperSignal West Femto (Thermo Fisher Scientific).

### NET-Associated Products Quantification

Isolated neutrophils were seeded at a concentration of 2 × 10^6^ cells/ml, stimulated with 50 nM PMA, washed, digested with S7 nuclease (Cayman Chemicals) for 20 min, centrifuged and stored at −20°C. Neutrophil elastase, myeloperoxidase (both from Abcam), LL37 (Hycult Biotech, Wayne, NJ, United States) and DNA-histone associated complex (Sigma-Aldrich, St. Luis, United States) concentrations were evaluated by ELISA. The DNase I concentration in the human serum was also determined by ELISA (LifeSpan BioSciences, Seattle, WA, United States).

### T Cell Induction

Peripheral blood mononuclear cells at a concentration of 10^6^ cells/ml were co-cultured with 1,000 ng/ml of autologous NET fragments for 7 days. On day 7, IFNγ- and IL-17-producing T cells were stained according to a previously published protocol (22). For T regulatory lymphocyte determination, we used a previously published staining protocol (23).

### Generation of Monocyte-Derived Dendritic Cells and Co-culture With NETs

Monocyte-derived DCs were generated from adherent monocytes according to a protocol published previously (24). Immature dendritic cells (iDC) were seeded at a concentration of 10^6^ cells/ml with or without 1,000 ng/ml NETs from the same donor. After 24 h, moDCs were stained with antiCD11c-APC and CD14-PEDy590 (Exbio), CD80-FITC (Beckman Coulter) as well as CD86-PECY5, HLA-DR-PECY7 (both from BD Bioscience), PDL1-PE (BioLegend) and DAPI (Thermo Fisher Scientific). The viability of the moDCs was estimated as DAPI negative for CD11c positive moDCs. The samples were collected using a BD FACSAria II (BD Biosciences), and BD FACSDiva software (BD Biosciences) was used for signal acquisition.

### moDC-T Cell Cultures

The capacity of iDC and iDC-NETs to activate T lymphocytes was evaluated with autologous DC-T cell cultures. The assays were carried out in media containing 5% human AB serum for 7 days at a 1:5 DC:T cell ratio. IL-2 (20 U/ml) was added on days 2 and 5. On day 7, Treg expansion and IFNγ and IL-17 production was analyzed as described in section T cell induction.

### Cytokine Production

For the detection of cytokine production, 10^6^ cells/ml moDCs were cultured in a 96-well plate and stimulated with autologous 1,000 ng/ml NETs for 24 h and the cytokine levels in the culture supernatants were determined by multiplex Luminex cytokine bead-based immunoassay (Millipore, Bedford, United States).

### L-Lactate Quantification

The concentration of L-lactate in PBMC and moDC culture supernatants was measured with a glycolysis cell-based assay kit (Cayman Chemicals).

### RNA-Seq

10^6^ moDC/ml from HD (healthy donors) were cultivated with 1,000 ng/ml of autologous HD-NETs, with T1D-NETs or left untreated for 8 h. Similarly, 10^6^ moDCs/ml from T1D were also cultivated with 1,000 ng/ml of autologous T1D-NETs, HD-NETs or left untreated for 8 h. The cells were then washed and lysed using RLT buffer (Qiagen, Hilden, Germany). Total RNA was isolated using RNeasy Mini Kit following manufacturer’s instructions (Qiagen), RNA quality and quantification was determined by TapeStation 4200 (Agilent, St. Clara, CA, United States) following manufacturer’s instructions.

Library construction was conducted employing the Illumina True Seq stranded mRNA Poly A protocol and subsequently sequenced using a HiSeq 4,000 (1 × 50bp single end reads, 15 samples per lane) following the manufacturer’s guidelines. This resulted in approximately 21 million reads before and 17 million reads per sample after mapping. RNAseq reads were trimmed for adapters using cutadapt (version 1.8.1) (25) while low quality signals were removed using the fastq illumina filter^1^. Resulting reads shorter than 50bp were omitted from further analysis. Subsequent mapping to the human reference genome GRCh38 was performed using TopHat (version 2.0.14) (26). Gene expression counts were quantified using HTSeq (27). Differential expression was determined by employing a Mann-Whitney-U test, followed by estimating a false discovery rate based on a Westfall & Young permutation (with *k* = 5,000 permutations) as previously published (28). Only genes with a *p*-value ≤ 0.05 and a FDR ≤ 5% were considered differentially expressed. Data is available from NCBI Gene Expression Omnibus (GEO) with accession number GSE143143.

### Statistical Analysis

The results obtained from at least three independent experiments are given as the means ± SDs. Not all patients were involved in all experiments due to the limited amount of blood available per sample. Statistical analysis was performed using non-parametric one-way analysis of variance (ANOVA) with multiple comparisons Dunn’s post-test where applicable. A two-tailed paired Wilcoxon or unpaired Mann-Whitney *t*-test was also applied for data analysis using GraphPad Prism 8. Values of *p* < 0.05 (^∗^), *p* < 0.01 (^∗∗^) *p* < 0.001 (^∗∗∗^) and *p* < 0.0001 (^****^) were considered statistically significant.

## Results

### The Presence of T1D Patient NETs Induces IFNγ-Producing T Cells *in vitro*

To estimate the biologic impact of NETs on T cells, the canonical inducers of T1D, we co-cultured PBMCs with 1,000 ng/ml autologous NET fragments and then evaluated the delineation of T cell populations into Th1 cells (IFNγ-producing CD4 T cells), Th17 cells (IL-17A-producing CD4 T lymphocytes), T regulatory lymphocytes (Treg, FoxP3^+^CD25^*hi*^CD127^–^ CD4 T cells) and IFNγ-producing CD8 lymphocytes. The gating strategies are illustrated in Supplementary Figures 1A, B. We obtained NET fragments by stimulating neutrophils isolated from the peripheral blood of T1D patients and healthy control donors with PMA for 3 h, cleaved the NETs with a restriction enzyme mix and used the components consisting of large DNA fragments (Supplementary Figure 1C) and NET-associated proteins, such as proteinase 3 (PR3) (Supplementary Figure 1D), for co-cultures and further composition analysis.

**FIGURE 1:**
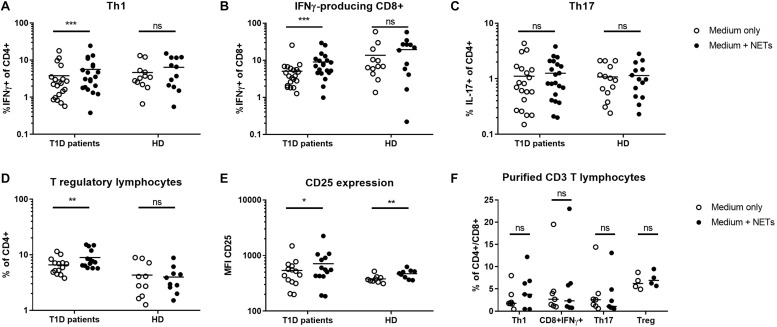
Shift in T lymphocyte populations. Populations of **(A)** Th1 (IFNγ+CD4), **(B)** IFNγ-producing CD8+ lymphocytes, **(C)** Th17 (IL-17+CD4), **(D)** T regulatory lymphocytes (Tregs) (CD4+FoxP3+CD25^*hi*^CD127-) with **(E)** CD25 expression on their surface were analyzed by flow cytometry in cultures from 20 T1D patients (55% female) and 12 healthy donors (50% female) after 7 day cultivation with or without autologous NET fragments. **(F)** Induction of Th1, Th17, Treg, and IFNγ+CD8 T cells isolated CD3+ lymphocytes co-cultured with or without autologous NETs for 7 days in T1D patients (*n* = 7) evaluated by flow cytometry. Statistical analysis was performed using a two-tailed Wilcoxon paired *t*-test and an unpaired *t*-test. Values of *p* < 0.05 (*), *p* < 0.01 (**) and *p* < 0.001 (***) were considered statistically significant.

T1D patients NETs induced a strong shift toward IFNγ-producing CD4+ and CD8+ cells in autologous T lymphocytes, which was also accompanied by an increase in Tregs but not IL-17A-producing Th17 lymphocytes (Figures 1A–D). In the control culture where healthy cells were treated with their own healthy NETs, there was no significant enrichment of any of these populations, but expression of CD25, an important marker of Treg function, was increased on Tregs after co-cultivation with NETs from both T1D patients and healthy donors (Figure 1E).

To clarify the influence of other cellular populations, such as professional antigen presenting myeloid (mDC) or plasmacytoid dendritic cells (pDC) and monocytes, on T cell delineation, we isolated CD3+ cells from PBMCs and co-cultivated them with autologous NETs for 7 days. We did not observe any significant changes in T cell populations under these conditions (Figure 1F), suggesting a role for dendritic cells and monocytes in mediating the biological effect of NETs in T1D.

### Monocyte-Derived Dendritic Cells of T1D Children Are Uniquely Responsive to NETs

**FIGURE 2:**
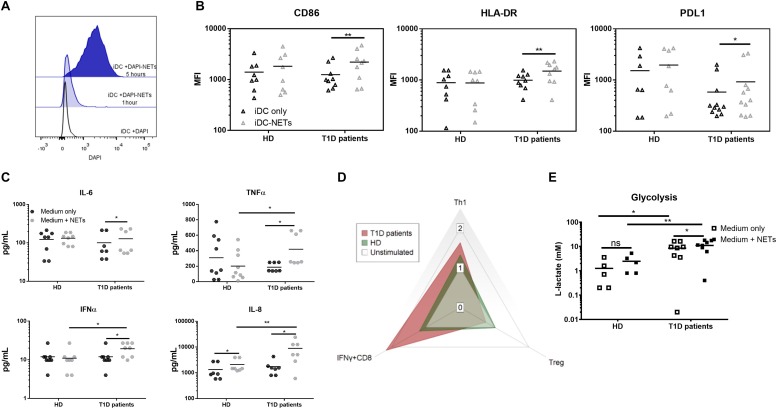
Monocyte-derived dendritic cell functional analysis. **(A)** Representative histogram of the interaction between moDCs and autologous NETs dyed with DAPI. **(B)** CD86, HLA-DR and PDL1 expression in the moDC co-cultured with or without autologous NET from 9 T1D patients (67% female) and 8 healthy donors (62% female) detected by flow cytometry. **(C)** Cytokine production by moDCs from 7 T1D (57% female) and 8 healthy (62% female) donors co-cultured with or without autologous NET analyzed by Luminex. **(D)** Radar graph displaying a shift between T cell populations induced by moDCs co-cultured with or without autologous NET. Data are expressed as the median of the response index (% of cells cultured with NETs/% of cells cultured without NETs). T cell populations analyzed by flow cytometry are defined as Th1 (IFNγ+CD4), Treg (FoxP3+CD25^*hi*^CD127-CD4) and IFNγ-producing CD8+ lymphocytes. **(E)** moDCs of 8 T1D (62% female) and 5 healthy (60% female) donors were incubated with or without autologous NETs for 24 h, and then the L-lactate concentration in the culture supernatant was measured as an indicator of glycolytic activity by ELISA. Statistical analysis was performed using a two-tailed Wilcoxon paired *t*-test and an unpaired *t*-test. Values of *p* < 0.05 (*), *p* < 0.01 (**) and *p* < 0.001 (***) were considered statistically significant.

Due to the rarity of dendritic cells in peripheral blood, elucidating their role in response to NETs required us to adopt the monocyte-derived dendritic cells (moDCs) model. We thus generated moDCs of healthy donors, as well as of T1D patients from adherent monocytes. On day 6, immature dendritic cells (iDCs) were harvested and matured by co-cultivation with NET fragments, and interactions became apparent in some cells within 1 h of co-culture (Figure 2A) and universal for all iDCs within 5 h.

We observed no change in the iDC phenotype after 24 h of autologous NET co-culture in healthy donors. On the other hand, patient iDCs significantly upregulated their surface expression of CD86, HLA-DR and surprisingly also PDL1 (Figure 2B). This upregulation was mirrored by a significant increase in IL-6, TNFα and IFNα production (Figure 2C), as well as an increased glycolytic activity present in T1D but not healthy iDCs (Figure 2E) co-cultured with NETs.

We verified the functional impact of these changes by co-culturing the healthy as well as patients’ NET-matured moDCs with autologous T cells. Patient NET-moDCs induced a very strong IFNγ-producing T cells and fewer Tregs with significantly lower CD25 expression compared to that of the healthy NET-moDCs (Figure 2D and Supplementary Figures 1E–H).

### NETs in T1D Patients Differ in Composition and Are Rich in DNA-Histone Complexes

**FIGURE 3:**
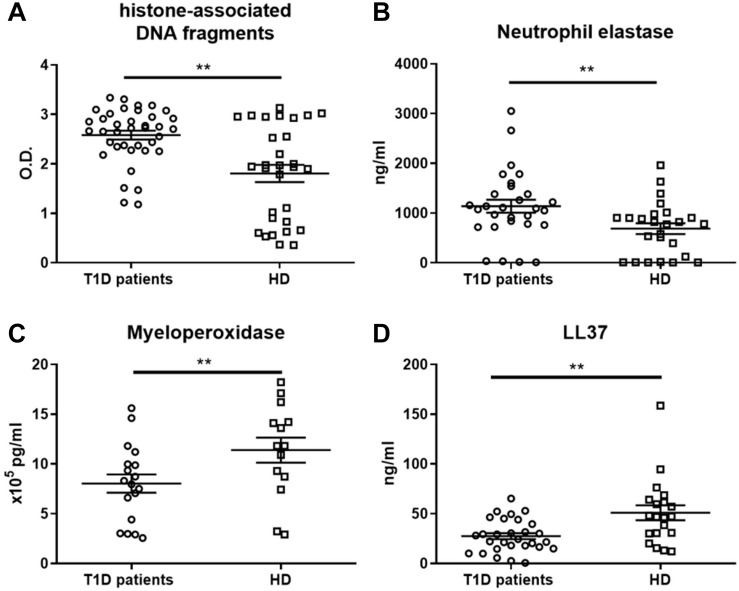
NET-associated product quantification in neutrophil extracellular traps. **(A)** Levels of histone-associated DNA fragments, **(B)** neutrophil elastase (NE), **(C)** myeloperoxidase (MPO) and **(D)** LL37 in NET fragments of T1D patients (56–65% female) and healthy donors (56–68% female), detected by ELISAs were analyzed in 14, 29 or 38 T1D patients and 14, 20 or 30 HD respectively. Statistical analysis was performed using unpaired *t*-tests. Values of *p* < 0.01 (**) were considered statistically significant.

To further characterize whether the observed proinflammatory profile of moDC after NET exposure in T1D patients was caused by properties of NETs or by inherently altered functions of patient moDCs, we investigated NET composition. The composition of NETs varied in the T1D patients from the healthy donors. Patient NETs contained significantly more DNA-histone complexes (Figure 3A) and neutrophil elastase (NE) (Figure 3B) but less myeloperoxidase (MPO) (Figure 3C) and LL37 (Figure 3D). Interestingly, when we tested DNaseI concentration in the serum of T1D patients and healthy donors as a marker of NET digestion and regulation of inflammation, T1D patients had higher DNaseI levels than did the healthy donors (Supplementary Figure 1I).

Overall, we observed a significant shift toward DNA-histones and NE and away from the NET-associated proteins MPO and LL37 in the T1D patient NETs, but we observed no correlation of NET composition with age, HbA1c, age at diagnosis, or T1D duration (data not shown).

### T1D NETs Can Induce a Diabetogenic Signature in Healthy moDC

Even though we observed that T1D NETs displayed altered composition, we proceed to further characterize potential inherently amended functions of patients’ moDCs. We stimulated healthy moDCs with heterologous T1D-NETs or with autologous HD-NETs and did the same for T1D-moDCs (stimulated with autologous T1D-NETs or with heterologous HD-NETs) and then analyzed their transcriptomic profile.

Firstly, we noted different gene profiles between unstimulated HD-moDC and unstimulated T1D-moDC (Supplementary Figures 2A,B) with 1568 DEGs (differently expressed genes). Pathway enrichment analysis performed with ENRICHR (29, 30) drawing on the HumanCyc (31) curated database revealed that the highest enriched pathways include metabolic processes such as glycolysis or gluconeogenesis (Supplementary Figure 2D). Further dysregulation in the immune system and in particular signal transduction was revealed by Reactome (Supplementary Figure 2C).

**FIGURE 4:**
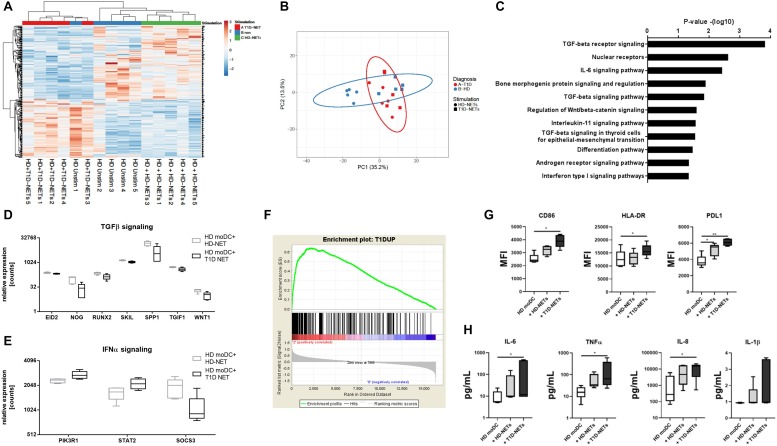
Analysis of RNA-Seq data from HD moDCs stimulated with autologous NETs versus HD moDCs stimulated with heterologous T1D NETs. **(A)** Cluster analysis of HD unstimulated moDCs, stimulated with autologous NETs and heterologous NETs. **(B)** principal component analysis (PCA) of HD and T1D moDCs stimulated with autologous NETs or heterologous T1D NETs **(C).** WikiPathways analysis of enriched pathways involved in moDC after NET stimulation **(D)** and **(E)** relative expression of enriched genes involved in TGFβ and IFNα signaling **(F)** GSEA (gene set enrichment analysis) of created a T1D signature gene set containing 1568 DEGs that were differentially expressed between unstimulated healthy moDCs and unstimulated T1D moDCs, divided into those that were upregulated and downregulated in T1D compared to HD. moDC from healthy donor (*n* = 5) (40% female) were cultivated with autologous healthy NETs (HD-NETs), heterologous T1D-NETs or left untreated for 24 h and their **(G)** phenotype determined by flow cytometry and **(H)** cytokine production analyzed by Luminex were evaluated. Statistical analysis was performed using unpaired *t*-tests. Values of *p* < 0.05 (*) and *p* < 0.01 (**) were considered statistically significant.

Next, we compared healthy HD-moDCs stimulated with autologous HD-NETs against HD-moDCs stimulated with heterologous T1D-NETs. 302 genes were differentially expressed between those two conditions (Figures 4A,B), and these genes showed that HD-NET-stimulated HD-moDC cluster together with unstimulated HD-moDC, as opposed to T1D-NET-stimulated HD-moDC.

The Wikipathway analysis (32) showed the top dysregulated pathways included TGFβ and IL-6 signaling due to the involvement of genes such as *WNT1, EID2, SKIL, SPP1* and *TGIF1* (Figures 4C,D). Expression of TGFβ related genes was higher in HD-NET stimulated HD-moDCs, whereas stimulation with T1D-NETs upregulated genes involved in IFNα signaling (with involvement of genes *SOCS3, PIK3R1, STAT2*) (Figures 4D,E) indicating that T1D-NETs induce a loss of tolerogenicity and proinflammatory IFNα response. We further illustrate this proinflammatory diabetogenic activity of T1D-NETs with a PCA (principal component analysis) analysis of the 302 DEGs, which grouped T1D moDC samples with healthy moDC treated with T1D-NETs, whereas HD-NET-stimulated HD-moDCs formed a separate cluster (Figure 4B).

To prove that T1D-NETs indeed drive a shift in HD-moDCs toward the transcriptomic profile of moDC of diabetic patients we created a T1D signature gene set containing 1568 DEGs that were differentially expressed between unstimulated healthy moDCs and unstimulated T1D moDCs, divided into those that were upregulated and downregulated in T1D compared to HD. Gene set enrichment analysis (GSEA) revealed significant enrichment (*p* = 0.0175, enrichment score 0.64) of the T1D-upregulated genes in T1D-NET-stimulated HD-moDCs. When healthy moDCs were treated with their own healthy NETs, there was no significant enrichment of this gene set (*p* = 0.051 and enrichment score 0.56) (Figure 4F).

Additionally, we also analyzed changes in phenotype and cytokine production in healthy moDCs upon exposure to hetelogous T1D-NETs and found out that HD-moDCs expressed significantly higher level of CD86, HLA-DR, PDL1 and produced markedly more proinflammatory cytokines under these conditions (Figures 4G,H).

## Discussion

NETs have recently become a hot topic for research due to their proposed role in the development of autoimmune diseases. In our study, we investigated the biological impact of NETs in a complex model of immune reactions in T1D patients. A Th1-skewed T cell response has been traditionally shown in T1D (33, 34), but has not yet been linked to a specific innate biological activity. Here, we show that NETs from T1D patients but not healthy controls induce IFNγ-producing CD4 and CD8 T cells, supporting the notion that it may be the presence of NETs in pancreatic tissue that stimulate the well-established downstream cascade of adaptive immune cell activation (14).

Our data showed that this induction of IFNγ-producing T cells does not arise from the direct effect of NETs on T cells, but is mediated through other cell populations. In the search for the cells responsible for the observed effect we have expanded our findings by adopting the monocyte-derived DC model (moDC), which allowed us to explore the rare population of dendritic cells, the primary inducers of T cell activation. In contrast to healthy moDCs that showed no reaction to NETs (35, 36), T1D patient moDCs reacted to NET fragments vigorously through a significant increase in their maturation markers, inflammatory cytokine production including the secretion of IFNα, as well as by their capacity to induce IFNγ-producing T lymphocytes. The NET-stimulated T1D moDCs also switched to anaerobic glycolysis, as documented by the increase in L-lactate levels, further corroborating their proinflammatory state (37, 38). Together, these results show different qualities of DCs in T1D patients in their reactivity to NETs, which again might support the idea that the T cell pancreatic infiltration may be promoted via NET-driven DC activation.

Intrigued by the observed proinflammatory response of T1D moDC to NETs, we decided to investigate further whether the profile is caused by inherent moDC properties or by the quality and composition of NETs themselves. While substantial evidence has been gathered on hyperglycemia promoting steady state but decreasing inducible NET formation, which in turn impairs wound healing in both type 1 and type 2 diabetes (15–17), it is still unclear whether T1D patient-derived NETs are inherently different from those of their healthy counterparts. In this study we observed decreased levels of antimicrobial peptides, MPO and LL37 in T1D NETs and additionally uncovered a strong shift toward histone-associated DNA fragments compared to healthy NETs. Particular histone modifications are an increasingly recognized quality of NETs influencing both the process of NET release itself and NET effect on numerous diseases (39), and observed differences in NET composition in T1D patients might strongly influence their biological activity, including their contribution to T1D development and progression. Since NETs in general are a source of autoantigens (40) they may contain islet autoantigens in T1D patients, which in turn would be processed by dendritic cells and further elicit both specific B cell and T cell responses (41). Potential capture of T1D autoantigens by neutrophils and their further release in NETs potentiating diabetogenic immune response is an intruiquing, however, as of yet unproven possibility. T1D patients display minor but not negligible neutropenia (42). Together with diminished levels of important antimicrobial peptides in NET structures, T1D patients might exhibit worsen defense against infections.

However, NET composition is not alone in its impact on the proinflammatory response our study describes. T1D PBMCs are known to harbor a distinct expression pattern of genes related to inflammatory response, fatty acid biosynthesis, hydrolase activity, lipid peroxidation and cell metabolism (43–45), which we were able to confirm also in our experimental model. Of note, we derived a prodiabetogenic signature from genes that were differentially expressed in T1D moDCs and transcriptome analysis newly revealed that even though T1D moDCs already carry altered steady state properties, T1D NETs but not healthy donor-derived NETs are uniquely able to induce a this prodiabetogenic signature in healthy moDCs. Thus, our data agree with the current hypothesis that both intrinsic factors, such as altered metabolic state of cells, as well as altered effect of T1D NETs, have an important role in the induction of proinflammatory profile of T1D moDC.

In summary, we show that NETs from T1D patients have different composition compared to those of healthy donors and induce different response in immune cells illustrated here in a model of monocyte-derived dendritic cells as well as directly in *ex vivo* peripheral blood PBMCs. T1D NETs promote activation, phenotypic changes, increased cytokine release and metabolic shift toward glycolysis compared to healthy NETs, and their addition to cell culture also drives T cell polarization toward IFNγ-producing CD4 and CD8 cells. These findings support the important role of neutrophils and NETs in the pathogenesis of T1D and identify another critical connection between the innate and adaptive immunity in T1D pathogenesis. Thus, we suggest that formation of NETs may be involved in triggering a proinflammatory response in myeloid cells, which in turn initiate a T cell-mediated immune response. Our work provides the basis for future studies which will test whether the observed proinflammatory NET properties derived from T1D patients are also present prior to the disease onset. Such intrinsic abnormalities might thus establish the NETs as active players in T1D development. To our knowledge this is the first study describing the distinct protein content in T1D NETs.

## Data Availability Statement

All data generated during this study are included in this published article [and its Supplementary Information Files] and are also available from the corresponding author on reasonable request.

## Ethics Statement

Written informed consents were obtained from the patient’s guardians in accordance with the Declaration of Helsinki and according to the procedures established by the Ethical Committee of our institution. Written informed consents for publication were obtained from parents of the presented children.

## Author Contributions

ZP designed the study and experiments, performed experiments, analyzed the data, interpreted the results, and wrote the manuscript. IZ and PV performed the experiments. AK reviewed and edited the manuscript, and analyzed transcriptomic data. RH provided RNA-seq and analyzed transcriptomic data. ZS, LP, and SP provided the patient information and biological material and reviewed the manuscript. AS designed the study, reviewed, and edited the manuscript.

## Conflict of Interest

The authors declare that the research was conducted in the absence of any commercial or financial relationships that could be construed as a potential conflict of interest.

## Footnotes

1 https://emea.illumina.com/products/by-type/informatics-products/basespace-sequence-hub/apps/fastq-toolkit.html
