# Supplementary material for: Neutrophil Extracellular Trap Induced Dendritic Cell Activation Leads to Th1 Polarization in Type 1 Diabetes
Source: Front Immunol. 2020 Apr 14;11:661. doi: 10.3389/fimmu.2020.00661 (PMC7172866; doi:10.3389/fimmu.2020.00661)
Supplement: Supplementary file 1 [file Data_Sheet_1.PDF]

Supplementary Data

Neutrophil extracellular trap induced dendritic cell activation leads to Th1 polarization in type 1 diabetes

Short running title: DC response to NETs in T1D

Zuzana Parackova[1], Irena Zentsova[1], Petra Vrabcova[1], Zdenek Sumnik[2], Stepanka Pruhova[2], Lenka Petruzelkova[2], Robert Hasler[3,4], Adam Klocperk[1], Anna Sediva[1]

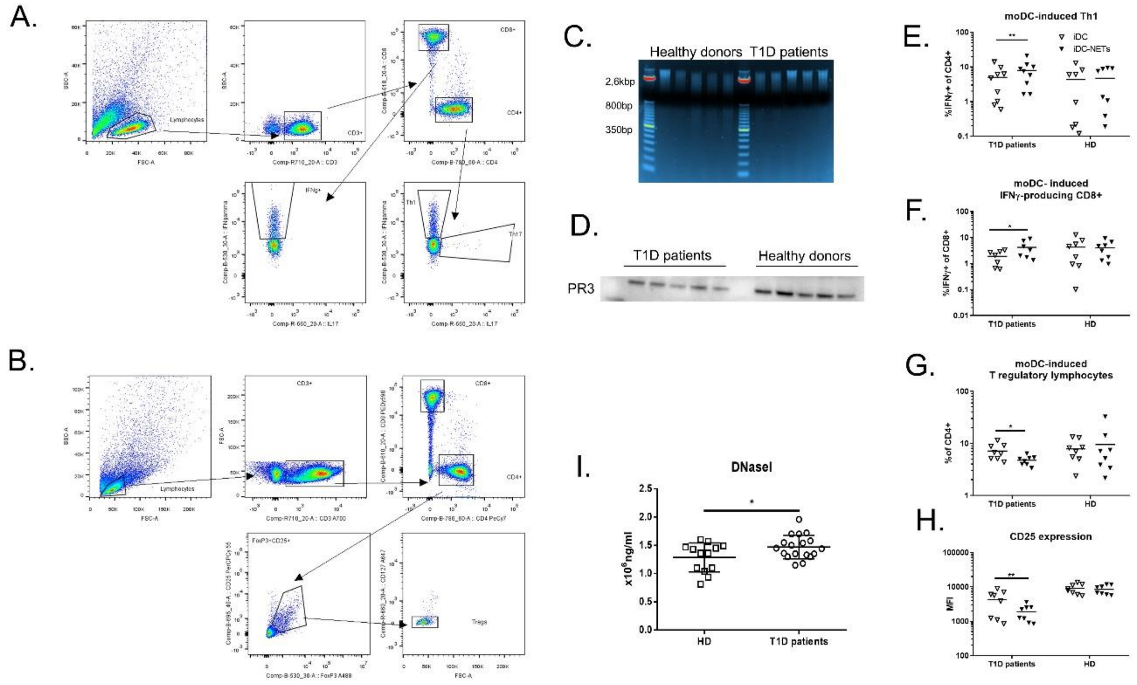

Supplementary Figure 1: Gating strategies for **A.** Th17, Th1 (IFNγ-producing CD4+), IFNγ-producing CD8+ lymphocytes and **B.** regulatory T cells. **C.** PMA-induced NETs were cleaved with a restriction enzyme mix and evaluated with gel electrophoresis for DNA fragment length and **D.** Western blot for containing proteinase 3. Monocyte-derived DCs cultivated with or without autologous NET fragments were cultivated with T lymphocytes for 7 days and induction of **E.** Th1 (IFNγ+CD4), **F.** IFNγ-producing CD8+ lymphocytes, **G.** T regulatory lymphocytes with **H.** CD25 expression on their surface from 9 T1D patients (67% female) and 8 healthy donors (62% female) were analyzed. **I.** DNAseI concentration in serum of 18 T1D patients (55% female) and 13 healthy donors (54% female). Statistical analysis was performed using a two-tailed Wilcoxon paired *t*-test and an unpaired *t*-test. Values of $p<0.05$ (*), $p<0.01$ (**) and $p<0.001$ (***) were considered statistically significant.

.

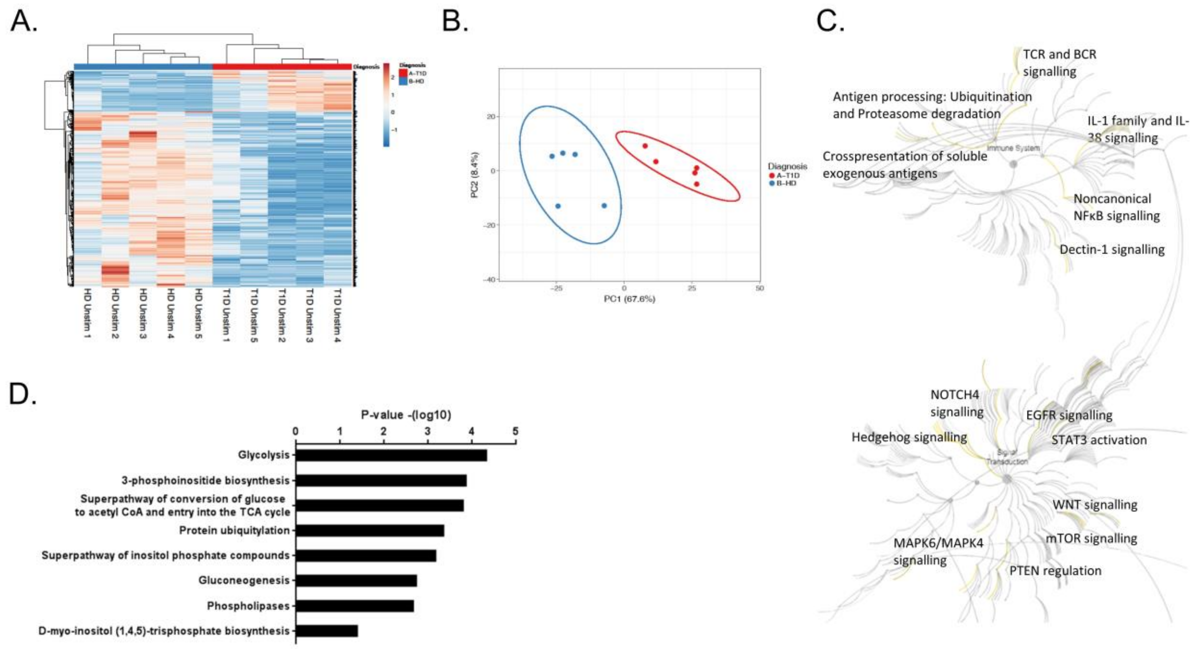

Supplementary Figure 2: Transcriptome analysis of unstimulated T1D moDCs and HD moDCs. A. Cluster analysis and **B.** principal component analysis (PCA) of T1D (80% female) and HD (40% female) unstimulated monocyte-derived DCs. **C.** Reactome analysis identified pathways that are enriched in moDCs. **D.** HumamCyc analysis of enriched pathways involved in moDC.
